# Nanoparticle-Laden Contact Lens for Controlled Ocular Delivery of Prednisolone: Formulation Optimization Using Statistical Experimental Design

**DOI:** 10.3390/pharmaceutics8020014

**Published:** 2016-04-20

**Authors:** Amr ElShaer, Shelan Mustafa, Mohamad Kasar, Sapana Thapa, Baljit Ghatora, Raid G. Alany

**Affiliations:** 1 Drug Discovery, Delivery and Patient Care (DDDPC), School of Life Sciences, Pharmacy and Chemistry, Kingston University London, Kingston upon Thames KT1 2EE, UK; k0846770@kingston.ac.uk (S.M.); d-kasar@hotmail.com (M.K.); k1104689@kingston.ac.uk (S.T.); B.Ghatora@kingston.ac.uk (B.G.); R.Alany@kingston.ac.uk (R.G.A.); 2 School of Pharmacy, The University of Auckland, Auckland 1142, New Zealand

**Keywords:** contact lenses, PLGA, nanoparticles, prednisolone, ocular drug delivery

## Abstract

Human eye is one of the most accessible organs in the body, nonetheless, its physiology and associated precorneal factors such as nasolacrimal drainage, blinking, tear film, tear turnover, and induced lacrimation has significantly decreased the residence time of any foreign substances including pharmaceutical dosage forms. Soft contact lenses are promising delivery devices that can sustain the drug release and prolong residence time by acting as a geometric barrier to drug diffusion to tear fluid. This study investigates experimental parameters such as composition of polymer mixtures, stabilizer and the amount of active pharmaceutical ingredient on the preparation of a polymeric drug delivery system for the topical ocular administration of Prednisolone. To achieve this goal, prednisolone-loaded poly (lactic-*co*-glycolic acid) (PLGA) nanoparticles were prepared by single emulsion solvent evaporation method. Prednisolone was quantified using a validated high performance liquid chromatography (HPLC) method. Nanoparticle size was mostly affected by the amount of co-polymer (PLGA) used whereas drug load was mostly affected by amount of prednisolone (API) used. Longer homogenization time along with higher amount of API yielded the smallest size nanoparticles. The nanoparticles prepared had an average particle size of 347.1 ± 11.9 nm with a polydispersity index of 0.081. The nanoparticles were then incorporated in the contact lens mixture before preparing them. Clear and transparent contact lenses were successfully prepared. When the nanoparticle (NP)-loaded contact lenses were compared with control contact lenses (unloaded NP contact lenses), a decrease in hydration by 2% (31.2% ± 1.25% hydration for the 0.2 g loaded NP contact lenses) and light transmission by 8% (unloaded NP contact lenses 94.5% NP 0.2 g incorporated contact lenses 86.23%). The wettability of the contact lenses remained within the desired value (<90 °C) even upon incorporation of the NP. NP alone and NP-loaded contact lenses both displayed a slow *in* *vitro* drug release of drug over 24 h; where 42.3% and 10.8% prednisolone release were achieved, respectively. Contact lenses can be used as a medicated device to sustain ocular drug delivery and improve patient compliance; nonetheless, patients and healthcare professionals’ acceptability and perceptions of the new formulations entail further investigations.

## 1. Introduction

Although the eye is one of the most accessible organs in human body, its physiology has made it quite impermeable to foreign substances. These characteristics protect the delicate structure of the eye from many harmful substances [1]. On the other hand, it has also proven to be a great challenge for ocular drug delivery purposes. Many approaches and strategies have been adopted to come up with an efficient delivery system to the eyes.

The most common dosage forms used for ocular drug delivery are eye drops, ointments, gels, or emulsions; they are mostly used to treat ocular surface and anterior segment diseases. It is the most preferred method due to the ease of drug administration and low cost. The site of action for most topically applied ophthalmic drugs is the cornea, conjunctiva, sclera, and the other tissues of the anterior segment such as the iris and ciliary body (anterior uvea). Following topical administration, several factors negatively affect the bioavailability of such formulations; these factors are known as precorneal factors and include nasolacrimal drainage, blinking, tear film, tear turnover, and induced lacrimation [2]. These factors cause the contact time to be drastically decreased, which is the primary reason why only 5% or even less of the applied dose reaches the intraocular tissues [3]. Hence, formulation scientists became interested in new alternatives for conventional ocular delivery systems. Ocular films, inserts and soft contact lenses were investigated, and some of them have managed to reach the market such as Ocusert^®^ and Lacrisert^®^.

Soft contact lenses are promising delivery devices that can sustain the drug release and prolong residence time by acting as a geometric barrier to drug diffusion to tear fluid. Besides, medicated contact lenses have a potential market as they are in use by more than 125 million people around the globe [3]. The possibility of using contact lenses for ocular drug delivery was first discussed by Sedlacek in the mid-1960s. Soaking a pre-prepared contact lens was employed for drug loading in early studies with loading efficiency between 0.01 and 5.53 mg/lens [4]. Despite improving drug residence time, pre-soaked contact lenses failed to load sufficiently high quantities of therapeutic agents and failed to sustain the drug release. Nanoparticle-laden soft contact lenses were explored as nano-reservoirs to extend the drug supply across the eye. The study conducted by Jung and Chauhan, evaluated the release of timolol from nanoparticle-laden contact lenses. Ethylene glycol dimethacrylate (EGDMA) and propoxylated glyceryl triacylate (PGT) monomers were used to prepare the nanoparticles of 3.5 nm diameter. Around 48%–66% of timolol was loaded within the nanoparticulate systems and its release was sustained for 24–48 h in diffusion cells at a therapeutic dose without compromising the transparency of the contact lens [5]. A second study conducted by Fazly *et al.*, 2013 assessed the antimicrobial effects of silver nanoparticles loaded in contact lenses on *Pseudomonas aeruginosa* and *Staphylococcus aureus* over 72 h. Contact lenses with relatively high doses of 57.13 and 98.06 μg of silver nanoparticles managed to inhibit the growth of the pathogenic bacteria for 48 and 72 h [6].

There are several methods used to produce PLGA nanoparticles, the most common technique being emulsification-solvent evaporation. The method used to prepare the PLGA nanoparticles is likely to have an effect on the microstructure formed; one could end up with nanocapsules in which the drug is trapped inside the core or nanospheres where the drug is adsorbed at the surface. Furthermore, the drug release mechanism and *in vivo* performance of nanoparticle has been shown to be dependent on the polymer used and on the loading efficiency. This was demonstrated in a study by Fadel *et al.* (2010) where zinc (II) phthalocyanine (ZnPc) was encapsulated in PLGA nanoparticles and was compared to free ZnPc. The results showed that the encapsulated ZnPc resulted in considerably smaller tumor size than the free ZnPc indicating a successful drug delivery [7,8]. Moreover, Gupta *et al.* (2010) developed and characterized PLGA nanoparticles to load sparfloxacin for sustained delivery [9]. The drug-loaded nanoparticles were prepared using a precipitation method with different drug-to-polymer ratios. The size of the formed particles ranged between 180 and 190 nm and the zeta potential was −22 mV. The prepared batches with different drug-to-polymer ratio showed drug entrapment ranging from about 25% to 86%. Compared with the marketed formulation which released over 96% of the drug within 6 h, the PLGA nanoparticles sustained the drug release with about 85.8% release over 24 h [9].

Aksungur *et al.* (2011) formulated nanoparticles (NP) using PLGA and loaded cyclosporine A (CsA) [10]. NP were prepared using PLGA alone or in mixture with Eudragit^®^RL (positively charged polymer) or coated with Carbopol^®^ (hydrophilic, negatively charged and mucoadhesive). NP was formed using the oil in water emulsification method. High entrapment efficacy (91.3%) was demonstrated by PLGA nanoparticles (NP) loaded with CsA (93.4%) and also with a PLGA-Eudragit^®^RL mixture. These entrapment efficiency values were attributed to the lipophilic nature of CsA, which resulted in good solubility in the oil phase, thus preventing loss via the aqueous phase. The average particle size ranged between 148 and 219 nm for the Eudragit^®^RL-coated NP, whereas Carbopol^®^-coated NP had slightly larger particle size (393 nm). The drug release profile showed an initial burst release followed by slower release. The total drug released over 24 h was between 75% and 90% [10].

Prednisolone is a synthetic glucocorticoid derived from cortisol. Prednisolone acetate or sodium phosphate are used as ophthalmic suspensions in eye drops to reduce allergic reactions affecting the eye and to relieve the accompanying symptoms such as redness, swelling and itching of the eye; it was used in the current study as a model drug [11,12].

The main aim of this research is to investigate (using statistical experimental design) the parameters that will affect the formulation of PLGA nanoparticles for the ocular delivery of prednisolone. It is envisaged that the formed nanoparticles will be integrated into a soft contact lens to promote extended prednisolone release, hence improving ocular bioavailability.

## 2. Materials and Methods

### 2.1. Materials

Prednisolone (purity >98%, *M*_W_ 360.45) was purchased from Tokyo Chemical Industry (TCI) Co. Ltd., Tokyo, Japan. Poly dl-lactic-*co*-glycolic acid (PLGA) (lactide: glycolide (65:35), *M*_W_ 44,000–75,000) and polyvinyl alcohol (PVA; hydrolyzed 98%–99%) all were purchased from Aldrich Chemistry (Sigma-Aldrich) St. Louis, MO, USA. Dichloromethane (DCM), stabilized with 0.2% of ethanol was purchased from VWR Chemicals Hunter Boulevard, Leicestershire, UK. Sodium chloride (NaCl) provided from Fisher Scientific, Loughborough, UK (laboratory reagent grade, *M*_W_: 58.44); Sodium hydrogen carbonate (NaHCO_3_; laboratory reagent grade, *M*_W_: 84.01) provided from Fisher Scientific, Loughborough, UK; calcium chloride dehydrate (CaCl·2H_2_O; for molecular biology ≥99%, *M*_W_: 147.01) provided from Sigma Aldrich, Dorset, UK; Potassium chloride (KCl; ReagentPlusTM ≥99%, *M*_W_: 74.55) provided from Sigma Aldrich, Dorset, UK.

### 2.2. Prednisolone Quantification

Prednisolone was separated and quantified by liquid chromatographic analysis using (Shimadzu LC-2010A HT, Columbia, MD, USA) HPLC system. The separation was achieved through the use of Beckman Coulter Ultrasphere ODS column, Pasadena, CA, USA (5 μm, 4.6 mm × 25 cm). The mobile phase was composed of 60% methanol and 40% distilled water *v*/*v* and was pumped at a flow rate of 1 mL/min and an injection volume of 10 μL. Prednisolone had a retention time of 3.675 ± 0.007 min when analyzed at λ_max_ of 254 nm. The validation of the method was carried out in accordance with the International Conference of Harmonization (ICH) guidelines. For the detection limit, the calibration curve was established at a concentration range of 1–1000 μm with coefficient of variation (*r*^2^ = 0.999).

### 2.3. Design of Experiment (DoE)

Minitab Design of Experiment (DoE) software was used to investigate the variables affecting the preparation of PLGA nanoparticles. The following independent variables were identified and studied: (i) concentration of PLGA used ranging from 0.1% to 0.4% *w*/*v*; (ii) concentration of PVA used ranging from 4% to 7.5% *w*/*v*; (iii) concentration of prednisolone (API) to be incorporated in the nanoparticles ranging from 0.5 to 2.0 mg/mL; (iv) homogenization time in minutes ranging between 10 and 20 min. The composition of these batches is shown in Table 1.

### 2.4. Preparation of Prednisolone-Loaded Nanoparticles

Nanoparticles were prepared using the single emulsion-solvent evaporation method; the emulsion prepared was oil-in-water (O/W). Prednisolone was dissolved along with the PLGA in DCM, while PVA was dissolved in distilled water and was used in its aqueous phase [13,14].

The organic phase was slowly added to about one third of the aqueous phase while in a magnetic stirrer then stirred at 2000 rpm for three hours and left overnight at a slow speed of 500 rpm in the fume cupboard to allow evaporation of DCM. On the following day, the rest of the aqueous phase (remaining two thirds) was added to the emulsion and stirred again at 2000 rpm for 1 h. The dispersion was then sonicated in a water bath sonicator (Fisherbrand (FB-15049) sonicator, Fisher Scientific UK Ltd., Loughborough, UK) for 15 min. Then the formed emulsion was homogenized using a Copley homogenizer (model: Heidolph-DIAX 900, Sigma-Aldrich Company Ltd., Dorset, UK) at maximum speed for 10–20 min. After homogenization, samples were withdrawn to determine the particle size and the zeta potential of the formed nanoparticles. The samples were centrifuged using an ELMI centrifuge (Model: CM-6MT, ELMI Ltd, Calabasas, CA, USA) at 3500 rpm at 4 °C, for 2 h and the supernatant was collected to determine the drug-loading capacity of the nanoparticles.

### 2.5. Preparation of Nanoparticle-Laden Soft Contact Lenses

The NP-impregnated contact lenses were formulated once the results from the Minitab software were analyzed and the nanoparticles were optimized accordingly. Contact lens matrix was prepared with the following composition: HEMA (2-hydroxymethacrylate) (80%), MAA (methacrylic acid) (19%) and EGDMA (ethylene glycol dimethacrylate) (1%). The three-polymeric mixture was stirred until completely miscible. Specified amounts were then injected into casting molds to be thermally polymerized at 80 °C.

### 2.6. Nanoparticle Characterization

The particle size distribution and the zeta potential of the produced nanoparticles were determined using laser diffraction particle size analyzer (DLS; Malvern Instruments zetasizer Model: 3000 HSA, Malvern Instruments Ltd, Malvern, UK). Samples were adequately diluted and analyzed at 25 °C and at an angle of 90 °C and results were presented at mean values ± (SD), *n* = 3.

#### 2.6.1. Encapsulation Efficiency Measurement

HPLC method was used to determine the percentage (%) of drug encapsulation efficiency in the prepared nanoparticles. First, the nanoparticles were centrifuged (ELMI Model: Skyline CM-6MT, ELMI Ltd., Latvia), the supernatant was collected and filtered using syringe filters and analyzed with HPLC; Equation (1) was used to calculate% prednisolone encapsulation efficiency.

(1) $$Prednisolone\;encapsulation\;efficiency\;\left(\%\right)=\;\frac{\left(total\;amount\;of\;drug-free\;amount\;of\;drug\right)}{\left(total\;amount\;of\;drug\right)\times 100}$$

#### 2.6.2. Scanning Electron Microscopy (SEM)

Scanning electron microscope (SEM, Zeiss Evo50-Oxford instrument, Cambridge, UK) was used to study the surface morphology of the formed prednisolone nanoparticles. Samples were prepared by sprinkling prednisolone or adding a drop of nanoparticle suspension onto specimen stubs. After drying the suspension, stubs were loaded onto a universal specimen holder. In order to enable electricity conduction, samples were coated with a fine layer of gold using a sputter coater (Polaron SC500, Polaron Equipment, Watford, UK) at 20 mA for three minutes at low vacuum and in the presence of argon gas (Polaron Equipment, Watford, UK).

#### 2.6.3. Thermal Analysis

Differential Scanning Calorimeter (Mettler Toledo instrument (Model: DSC822^e^-, Mettler-Toledo Ltd., Leicester, UK) was used to characterize the solid state, polymorphism and phase behavior of the API and nanoparticles. Approximately 2–3 mg of the sample was weighed and placed into an aluminum sample pan (50 μL). An empty pan was used as a reference, both pans were covered with the aluminum lid and a small hole was pierced. The sample was then heated to 250 °C at a constant heating rate of 10 °C/min. Each sample was investigated in triplicates. The thermograms were analyzed using STAR^e^SW 10.00 software.

Thermogravimetric Analysis (TGA) was used (Mettler Toledo instrument, Model: TGA/DSC 1 STAR^e^ system, Mettler-Toledo Ltd., Leicester, UK). In this technique the changes in both the physical and chemical properties of the API and prepared nanoparticle are measured as a function of increasing temperature 25–300 °C with constant heating rate of 10 °C/min the decomposition pattern is observed.

#### 2.6.4. Freeze-Drying (Lyophilization)

Prior to freeze-drying, the nanoparticle suspension was put in a round bottom flask and was kept in dry ice for 12 h. The sample was freeze-dried so the water within the sample is removed by sublimation and desorption under vacuum (−50 °C). Nanoparticles were lyophilized for 24 h using a Telstar freeze dryer (Model: Cryodos, Dewsbury, West Yorkshire, UK).

### 2.7. Nanoparticle-Loaded Soft Contact Lens Characterization

#### 2.7.1. Contact Lens Preparation

The hydrogels were prepared using 2-hydroxyethyl methacrylate (80%) (HEMA) as a backbone monomer, methacrylic acid (19%) (MAA) as a functional polymer, ethylene glycol dimethacrylate (1%) (EGDMA) as a cross-linker agent and finally prednisolone as the drug. The hydrogels were synthesized by thermal polymerization at 80 °C for 4 h in polypropylene molds.

#### 2.7.2. Contact Lens Hydration

Following polymerization, the formed lenses were subjected to hydration testing by soaking in distilled water for 24 h at room temperature. Both dry and wet weights were recorded and the results were presented as mean values ± (SD), *n* = 3. The equation below was used to calculate the% hydration.

(2) $$Hydration\;\left(\%\right)\;=\;\frac{Lens\;wet\;weight\;–\;Lens\;dry\;weight}{Lens\;wet\;weight}\;\times \;100\;$$

#### 2.7.3. Surface Contact Angle

**Figure 1:**
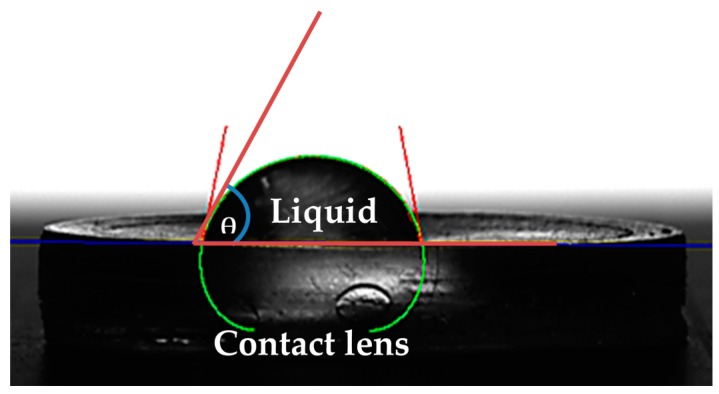
Measurement of contact angle is purely dependent on a series of connections recognized by Young (1805), where by the contact angle of a liquid drop on the solid surface is defined by the mechanical equilibrium of the liquid drop under the process of three tensions. This is defined as Young’s Equation (3).

Contact angle measurements were performed using KRUSS DSA30S, (KRÜSS GmbH, Borsteler Chaussee, Germany). The contact angle was measured using the static sessile drop method, after vertically dispensing droplets of deionized water of a specified volume (8 μL) onto the contact lens surface; using a high-tech optical camera, the angle that was created between the baseline of the drop (solid-liquid interface) and the tangent (liquid-air surface) was determined (Figure 1).

(3) σs = Ysl + σl ·cos θ

According to Young, there is a connection between the contact angle θ, the surface free energy of the solid σ_s_, the interfacial tension between the liquid and the solid Y_sl_, and the surface tension of the liquid σ_l_.

#### 2.7.4. Light Transmission

The transparency of all contact lenses was measured using UV-spectrophotometer at the wavelength of 600 nm [15]. The contact lenses were soaked in distilled water overnight making them flexible to fit into the cuvette after which light transmission was measured.

#### 2.7.5. Texture Analysis

TA.XT.plus Texture Analyser (Stable Microsystems Ltd, Surrey, UK) was used to determine the elasticity and tensile strength of the contact lenses. Hydrated contact lenses were cut into specific dimensions where length, width and thickness of the lenses were 25, 15 and 2 mm, respectively. The cut sample was then fit onto the texture analysis clamp and stretched until breaking point. Young’s modulus was calculated using the stress and strain values obtained. Tensile strength of the contact lenses was determined by using the maximum force applied until the breakpoint was reached. Equations (3) and (4) were used to calculate Young’s modulus and tensile strength, respectively. All measurements were carried out in triplicate and presented as mean value ± standard deviation.

(4) $$Young^{\prime }s\;modulus\;=\;\frac{Stress\;\left(MPa\right)}{Strain\;\left(\%\right)}$$

(5) $$Tensile\;strength\;=\;\frac{Force\;\left(N\right)}{Cross-sectional\;area\;\left(mm2\right)}$$

#### 2.7.6. Drug Release Study

An *in vitro* drug release study was carried out over 24 h using phosphate buffered saline (PBS) as the release medium. The contact lenses were immersed in 10 mL of PBS and placed in a mechanical shaking bath. The release of prednisolone from the NP alone was also detected by placing 0.4 g of NP in a dialysis bag and placing this bag in a plastic tube containing 10 mL of PBS. This tube was also placed together with the contact lenses in the shaker which was set at 80 cycles/min at 35.5 °C. Aliquots of 0.5 mL were taken at set time-intervals (0.5, 1, 1.5, 3, 6 and 24 h) and replaced by equivalent volumes of fresh PBS. Withdrawn samples were diluted with 2 mL of methanol, then run through HPLC to quantify concentration of prednisolone released.

#### 2.7.7. Statistical Analysis

All experiments were carried out in triplicate and the values were expressed as mean ± SD. Statistical data analyses were conducted using Minitab^®^ (Minitab, Inc, State college, PA, USA), one-way analysis of variance (ANOVA) was used and data generated were statistically significant with *p* < 0.05.

## 3. Results

### 3.1. Design of Experiment (DoE)

Two-level full factorial design was used to optimize PLGA nanoparticulate systems. Four independent variables (PLGA, PVA, API concentrations and homogenization time) were explored; once 16 experiments were generated by Minitab, the nanoparticles were formulated and evaluated based on three dependent variables (responses): particle size, encapsulation efficiency and surface charge. The DoE was conducted to optimize the formulation of NP and assist in identifying optimal conditions, namely; a small particle size (100–250 nm), maximum surface charge for higher stability and maximum encapsulation efficiency. Minitab was used to generate plots demonstrating the effects of each variable on the response.

### 3.2. The Effect of Different Parameters on Particle Size

**Figure 2:**
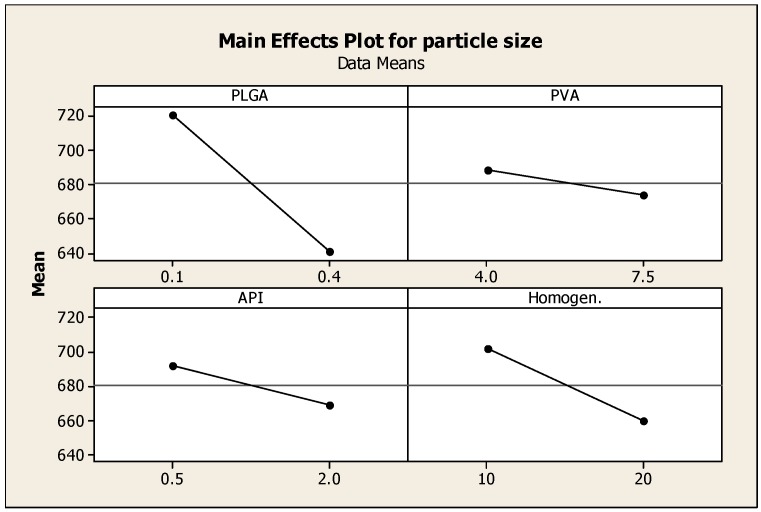
Main effects plot showing the effect of PLGA, PVA, prednisolone concentrations and homogenization time on the particle size of PLGA nanoparticles.

Particle size has a drastic effect on the bioavailability of drugs delivered using nanoparticles. Smaller particle size increases the surface area, hence the greater interaction with release medium; better solubility and improved dissolution. Figure 2 describes the effect of formulation parameters on particle size. Increasing the PLGA co-polymer concentration has the most predominant effect on decreasing the particle size. The samples that contained the highest concentrations of PLGA have resulted in the smallest nanoparticles, as shown by samples 4, 8 and 11. The amounts of PVA and API have resulted in a decreased particle size but was not as significant as the effect of increasing PLGA; increasing homogenization time also decreased particle size. Longer homogenization time along with higher amount of API (such as those used such in sample 11) yielded the smallest size of nanoparticles (538.8 nm). Many studies have demonstrated that both homogenization time and speed significantly affect the size of the formed nanoparticles. Javadzadeh *et al.* (2010) prepared PLGA nanoparticles loaded with naproxen; the study evaluated the effect of various homogenizing speeds on the formation of nanoparticles. Particles with a size range between 352 and 571 nm were formed; the smallest particle size was obtained by homogenizing at 20,000 rpm [16]. A more recent study by Narayanan *et al.* (2014) investigated the effect of process variables on the preparation of hyaluronidase-loaded PLGA nanoparticles. The effect of PLGA concentration, PVA concentration, internal and external phase ratio, homogenization speed and time on the mean particle, and zeta potential were studied. Results indicated that an increase in PVA concentration within external phase preparation contributed to smaller particle size of <400 nm, zeta potential of <−30 mV; this was ascribed to the surfactant stabilizing effect in reducing the surface tension of the continuous phase. Homogenization speed and time had maximum effect on reducing particle size [17,18].

### 3.3. The Effect of Formulation Parameters on Encapsulation Efficiency

**Figure 3:**
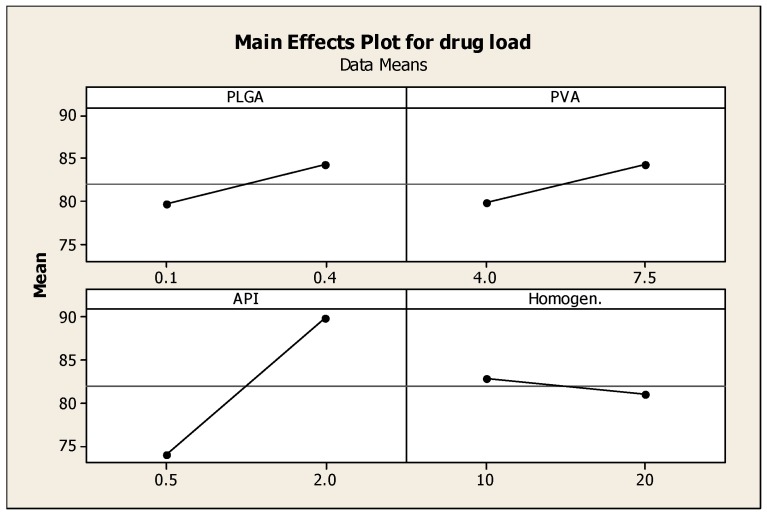
Main effects plot showing the effect of the four independent variables; PLGA, PVA, prednisolone concentrations and homogenization time on the drug loading into the nanoparticles.

Drug encapsulation efficiency of the nanoparticles was determined using HPLC. Drug encapsulation efficiency (EE) values ranged between 60% and 92%. These relatively high values of EE could be attributed to the use of dichloromethane, which is a very good solvent for the polymers and the drug employed, where all components were dissolved in this solvent medium (Blanco *et al.*, 2011) [19]. Figure 3 reveals that the concentration of prednisolone is the determinant factor of the drug entrapment efficiency. Higher drug-loading into the nanoparticles was obtained with samples 4, 9, 10, 11, 12, 13, 15 and 16, where the loading efficiency ranged between 85% and 95%. As shown in Figure 3 increasing the concentration of prednisolone (API) had the most significant effect in increasing the entrapment efficiency of the API in the formed nanoparticles, also increasing the amounts of PVA and PLGA had an effect on the entrapment efficiency but not as significant as the effect of API concentration; increasing homogenisaation time had a little or no effect on the entrapment efficiency. Improved entrapment efficiency may protect the API from enzymatic degradation, and could allow better API delivery to target site [19,20].

### 3.4. The Effect of Different Parameters on Surface Charge of Nanoparticles

**Figure 4:**
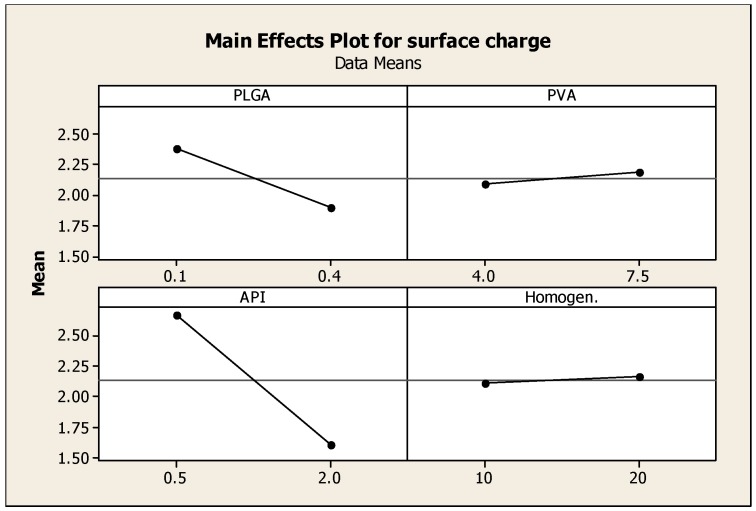
Main effects plot showing the effect of PLGA, PVA, prednisolone concentrations and homogenization time on the Zeta potential of prednisolone nanoparticles.

The values of the obtained surface charge ranged between 0.2 and 4 mV (Table 1, Figure 4). Low values of zeta potential reflect the formation of neutral nanoparticles; surface charge or the electrical charge properties control interactions between nanoparticles and therefore determine the overall behavior and stability of the nanoparticles [21].

### 3.5. Formulation Optimization

To achieve a particle size smaller than 500 nm, high surface charge and maximum drug entrapment efficiency, the following optimized conditions were identified by Minitab and used: 0.4% (g/100 mL) concentration of PLGA, 7.5% (g/100 mL) PVA, 2 mg/mL API (prednisolone), and 10 min of homogenization time.

### 3.6. Characterization of the Optimized Nanoparticle Preparation

The optimized prednisolone nanoparticles showed particle size of 294.5 ± 1.8 nm and surface potential of 5.6 ± 1.8 mV.

**Figure 5:**
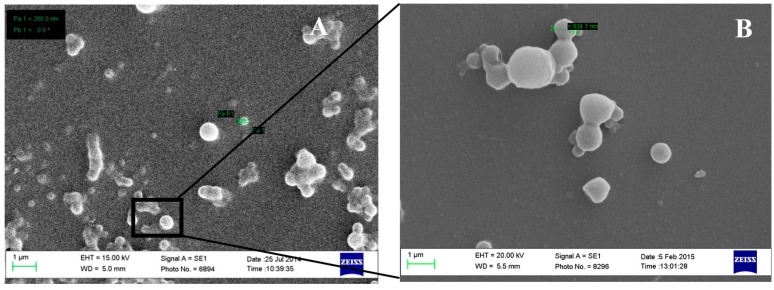
SEM of formulation (sample number pleased) showing individual and agglomerated nanoparticles at low (**A**) and high (**B**) magnifications.

The size and the surface morphology were elucidated using scanning electron microscope (SEM). Figure 5A shows individual and aggregates of prednisolone-loaded PLGA nanoparticles with particle size of around 300 nm. Aggregation could be attributed to the low surface charge carried by PLGA nanoparticles as suggested by the zeta potential studies. The prepared nanoparticles were spherical in shape with a smooth outer surface (Figure 5B).

### 3.7. Thermal Analysis

Differential scanning calorimetry (DSC) and thermogravimetric analysis (TGA) can be used to determine a number of characteristics such as physical and thermal properties by measuring the energy transfer of API, characterize the matrix solid state, including polymorphism and phase behavior.

**Figure 6:**
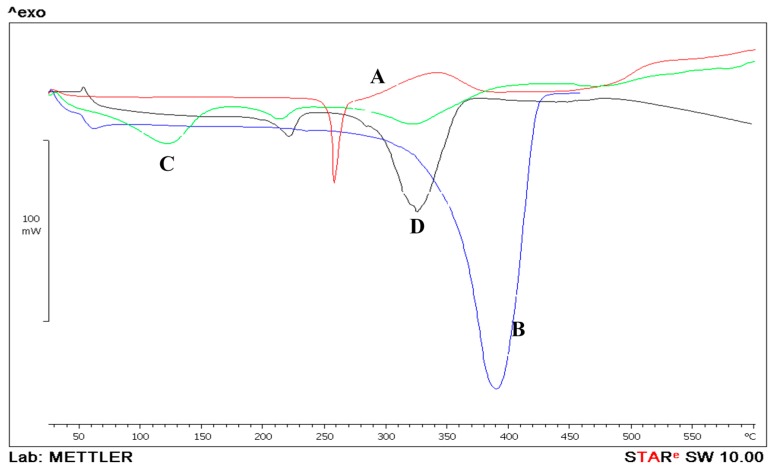
Differential scanning calorimetry thermograms of prednisolone (**A**); PLGA (**B**) and prednisolone-PLGA nanoparticles (**C**); PVA (**D**).

Figure 6 displays the DSC thermogram for the PLGA polymer, prednisolone and the prepared nanoparticles. Prednisolone exhibited an endothermic peak displaying melting at 250 °C [22]. PLGA had a major endothermic peak starting at around 342 °C that possibly could represent melting. The melting endotherm suggests the presence of some crystalline regions in PLGA polymer [23]. PVA showed a melting point at around 240 °C (Figure 6D) followed by a degradation at around 270 °C, similar results were suggested by [24]. PLGA NPs showed an endothermic peak at 120 °C which could be attributed to the evaporation of water that was used during the preparation of the nanoparticle. Coupling the DSC with the TGA data confirm the suggestion as the weight of the nanoparticles dropped by 11% as suggested by the TGA results. The second peak at 240 °C could be attributed to the presence of some PVA residue in the prepared NPs. On the other hand, the formulated PLGA nanoparticle did not show any major thermal events at 250 °C, suggesting a possible molecular dispersion of prednisolone within the PLGA polymer matrix during the formation of the nanoparticulate system (Figure 6B).

**Figure 7:**
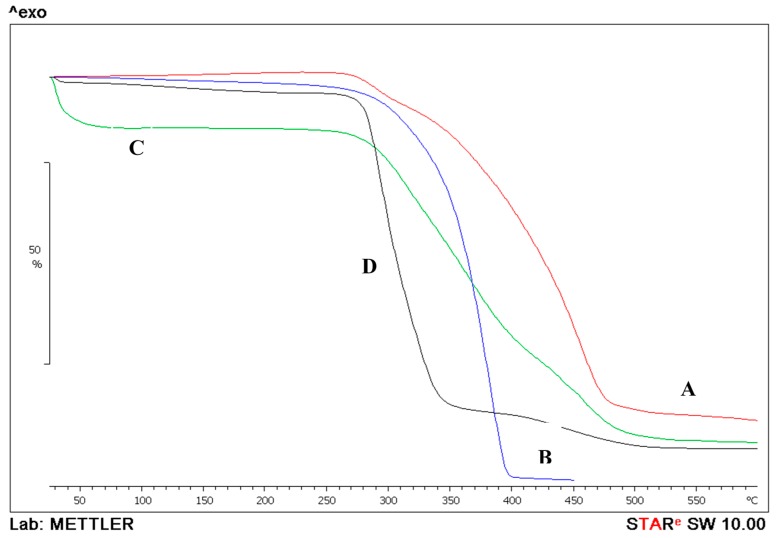
Thermogravimetric analysis of prednisolone (**A**); PLGA (**B**) and prednisolone-PLGA nanoparticles (**C**); PVA (**D**).

Figure 7 displays combined graphs of the thermogravimetric analysis of the PLGA, prednisolone and the prepared nanoparticles. PLGA by itself lost 98.7% of its mass by TGA in the endothermic event observed between 177.74 and 450.4 °C and a midpoint of 372.18 °C. Prednisolone also lost its mass in two events. First mass loss of 7.72% was between 209.5 and 313.57 °C with a midpoint of 291.21 °C. The second event was a loss of 78.26% of the remaining mass between 315.7 and 601 °C at a midpoint of 433.74 °C. When the prepared nanoparticles were tested by TGA, the midpoints were delayed and occurred at higher temperatures as the mass (3.4 mg) was lost gradually in three events. The first was at a midpoint of 71.6 °C where the mass loss of 11% could be attributed to the evaporation of any residual solvents; the second loss was at a midpoint of 343.6 °C, where the mass loss of 50.6% was similar to the midpoint of the prednisolone graph, proving that the API was successfully incorporated in the preparation. The mass lost in the third event was at midpoint 453.7 °C and was equal to 26.6% due to decomposition.

### 3.8. Nanoparticle Incorporation into Contact Lenses (Preparation and Characterization)

The optimized nanoparticles were incorporated in the contact lens matrix made of 80% HEMA, 19% MAA as a functional polymer cross-linked by 1% EGDMA. The formulated soft contact lenses were subjected to a number of characterization studies in order to understand their physical, optical and mechanical properties.

#### 3.8.1. Hydration and Surface Contact Angle

The ocular surface and anterior eye segment relies on the aqueous humor for nutrients and on external supply of oxygen through the precorneal tear film and cornea. Hence, it is very essential for ocular hydrogels or contact lenses to permit sufficient oxygen supply [25]. Good contact lens hydration is required to allow efficient oxygen permeability [26]. If lens hydration is too low, then an insufficient amount of oxygen would reach the cornea. This would lead to various conditions such as acute and chronic hypoxia. However, very high water content could render the contact lens prone to dehydration [27]. Extended wear of contact lenses could also cause discomfort to the eye as the hydration decreases over time leaving the eyes dry [28]. It is therefore important that contact lenses remain appropriately hydrated to avoid any future complications and discomfort for the users.

**Figure 8:**
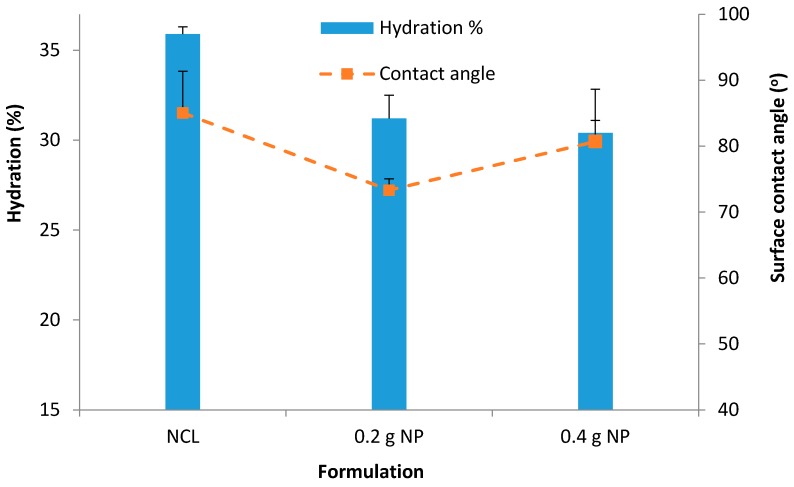
Hydration % for unloaded NP contact lenses, contact lenses with 0.2 g prednisolone nanoparticles and 0.4 g prednisolone nanoparticles. Data presented as mean + standard deviation, *n* = 3.

**Figure 9:**
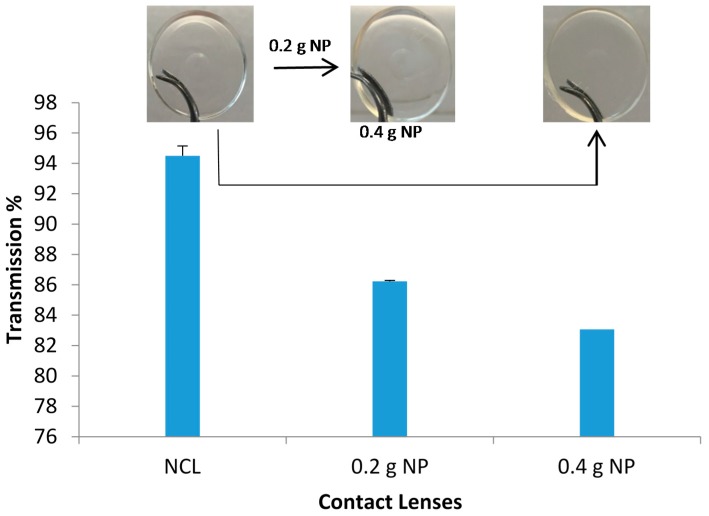
Light transmission through contact lenses at visible wavelength of 600 nm. Data are presented as mean ± standard deviation, *n* = 3).

The average hydration for unloaded NP contact lenses was found to be 35.9% ± 0.4% (Figure 8). Contact lenses with NP demonstrated a decrease in hydration compared with the unmodified (normal) contact lenses as follows; 31.2% ± 1.3%; *p* = 0.01 (0.2 g NP) and 30.4% ± 0.7%; *p* = 0.0005 (0.4 g NP) (Figure 9).

Figure 8 for hydration showed that there was a significant (*p* < 0.05) decrease of hydration with the addition of the NP. The decreasing pattern maintained with the addition of more NP; 31.2% ± 1.25% with 0.2 g NP (*p* = 0.01) and 30.4% ± 0.67% with 0.4 g NP (*p* = 0.0005). Similar decrease of water content with integration of NP was reported by Jung *et al.* (2012) [28], in which the authors reported a decrease (from 73% to 33.27%–63.82%) in the presence of NPs in the hydrogels depending upon the amount of NPs.

Surface wettability is very crucial for contact lens comfort. Good wettability of contact lenses helps maintain a normal and functional ocular surface. Poor surface wettability will increase lipid deposition and promote protein denaturation. Contact lenses investigated in this study all had contact angles below 90 °C which suggests good wettability (Figure 8). A lower contact angle was obtained upon incorporation of NP (*versus* 85 °C with unmodified (normal) lenses, 73.3 °C (*p* = 0.04) and 80.7 °C (*p* = 0.25) with the 0.2 g and 0.4 g NP-loaded contact lenses, respectively) (Figure 8).

The degrees of wettability of contact lenses are established based on the adhesive and cohesive forces. For lenses to be comfortable and devoid of eye dryness effect they must have a good wettability. The human eyes blink very frequently 12 blinks a minute on average, and with every blink, the tear film spreads over the ocular surface and helps to maintain hydration. Contact lenses with good wettability will not impair the precorneal tear film spreading onto the ocular surface [28]. The improved wettability of the NP-loaded contact lenses could be due to the contribution of the hydrophilic monomers (HEMA and MAA). It was seen that the addition of NP significantly lowered the contact angle.

#### 3.8.2. Light Transmission

Ideally, a soft contact lens should have light transmittance of above 90%; however some of the reported values in the literature for p-HEMA by itself was 87% [23]. Incorporation of nanoparticulate systems can drop the transparency of the contact lenses significantly as the nanosized particles diffract and scatter the incident lights. Gulsen *et al.* reported that incorporating microemulsions into p-HEMA contact lenses dropped light transmittance to 79%, 69% and even 4.4 and 19% [23].

The unloaded NP contact lenses prepared using the mixture of HEMA and MAA were all clear and transparent; their light transmission measured at 600 nm showed high transmission of 94.5% (Figure 9). The incorporation of NP did lower the transmission to 86.23% and 83.1% for the 0.2 g and 0.4 g NPs, respectively (Figure 9). It was observed that the increase in NP concentration gradually decreased the transmission. Therefore, it was necessary to add the appropriate amount of NP to avoid lack of transparency, which in turn would affect vision. These observations were comparable to previous studies which tested microemulsion-loaded hydrogels [29]. The results obtained here supported the use of NP in contact lenses without compromising the visibility. The study suggested that there was low or no opacity. The use of the contact lenses with suitable amount of NP would not affect the vision of the wearer which is essential. Compromised vision when using the contact lenses would result in poor compliance.

### 3.9. Mechanical Characterization

#### 3.9.1. Young’s Modulus

Contact lenses must possess good mechanical characteristics; otherwise they could be prone to damage during handling and insertion within the eye, and could be uncomfortable to wear [30]. The elasticity—given by the Young’s modulus—showed minimal changes to all of the lenses. A high proportion of hydrophilic monomers increase the water content. However, high water content can result in a weak mechanical strength of the material [30]. Therefore, a combination of HEMA and MAA was used to boost the hydrophilicity and also maintain mechanical strength. Low tensile strength and modulus for the contact lenses were observed and this could be attributed to the use of the hydrophilic copolymers. It should be noted that the stiffness of the lenses increases with the modulus of elasticity [30]. Hence, it can be said that the contact lenses formulated which demonstrated small Young’s modulus values only suggests a soft flexible material therefore more comfortable for long wear.

**Figure 10:**
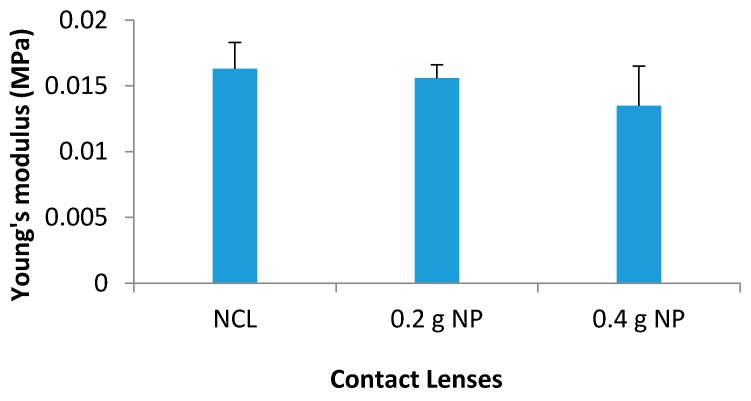
Young’s modulus (MPa) for all contact lenses.

Young’s modulus for NCL was 0.0163 ± 0.002 MPa. The addition of NP resulted in slight but non-significant decrease in the Young’s modulus. The average value for 0.2 g NP was 0.0156 ± 0.001 MPa (*p* = 0.314) and for 0.4 g NP was 0.0135 ± 0.003 MPa (*p* = 0.121) (Figure 10).

#### 3.9.2. Tensile Strength

The contact lenses were subjected to maximum load or stretched until a breaking point, *i.e.* the maximum force needed to achieve this breakpoint. This value—force at breakpoint—together with the cross-sectional areas of the contact lenses were used to calculate the tensile strength.

**Figure 11:**
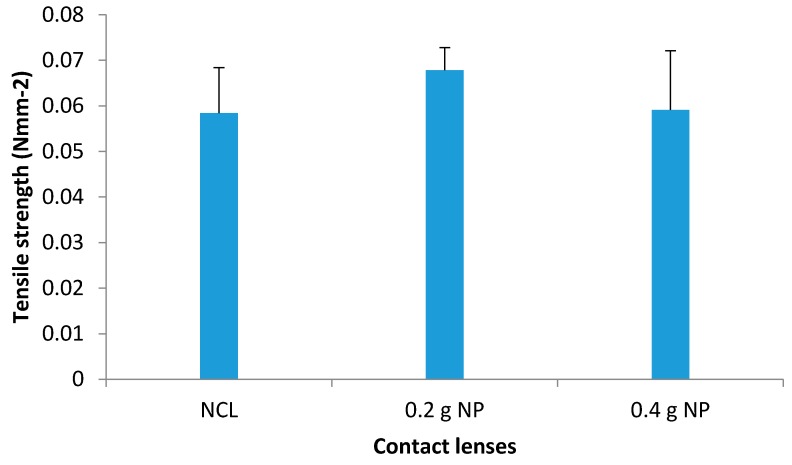
Tensile strength (Nmm^−2^) of NCL, 0.2 and 0.4 g prednisolone NP-loaded contact lenses (mean ± standard deviation, *n* = 3).

The tensile strength for NCL was calculated to be 0.0584 ± 0.01 Nmm^−2^ (Figure 11). The values did not change significantly with the addition of nanoparticles in the contact lenses (0.0678 Nmm^−2^; *p* = 0.137 for 0.2 g NP *vs.* 0.0591 Nmm^−2^; *p* = 0.470 for 0.4 g NP). These findings suggest that the contact lenses can maintain their elasticity even upon incorporation of 0.4 g of NP, thus corroborating the suggestion in a previous study by Tranoudis *et al* [28] of establishing a positive correlation between the elasticity and tensile strength.

#### 3.9.3. Drug Release Study

**Figure 12:**
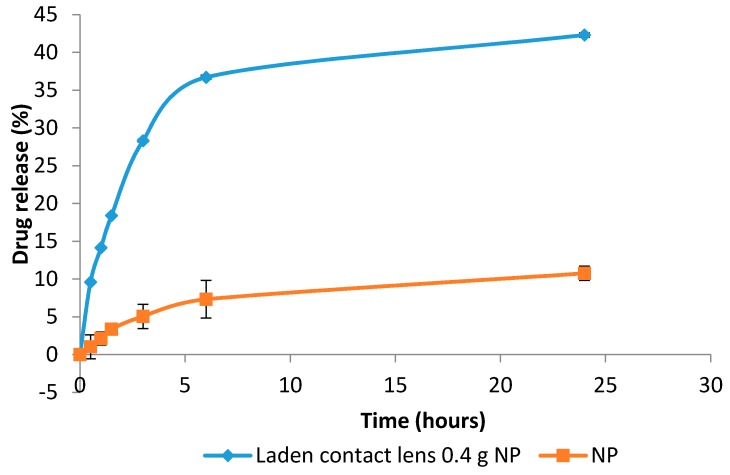
Drug release pattern of nanoparticles (NP) alone and contact lenses loaded with 0.4 g of nanoparticles over 24 h.

*In vitro* release of prednisolone from the NP-loaded contact lenses and NP on their own showed a two-phase process. An initial burst release was seen for both samples. This was then followed by a slower release process (Figure 12).

The drug release study was carried out over 24 h using 10 mL of PBS as the release media [22]. The contact lenses containing 0.4 g NP and the NP alone were subjected to this test. The total drug release over 24 h from NP and NP-loaded contact lenses were 42.3% and 10.8%, respectively. This could be ascribed to the fact that the drug needed to diffuse from both inside the NP and then the contact lenses matrix in case of NP-loaded contact lenses delaying the release. The initial release could be due to the entrapped drug attaching to the surface. The slow release is most likely to be associated with drug entrapped in NP [23]. For the drug to be released into the tear fluid and onto the ocular surface, it needs to diffuse through the polymeric matrix of the NP. PVA could also form a barrier due to its swelling ability which again contributed to a slower release [9].

It has been reported that ocular drugs marketed as eye drops have fast drug release whereby nearly all of the drugs were released within few hours [9]. The incorporation of drugs in NP extended the delivery by hours or even days [24]. A study by Gupta *et al.* tested the drug release of a commercially available formulation of sparfloxacin (an antibiotic) with PLGA-NP containing the same drug. The results showed that the commercial formulation released almost all drugs within 6 h, whereas the NP released about 85.8% drug in 24 h [9]. The data obtained in this work supported these findings regarding sustained release by maintaining a slow drug release.

## 4. Conclusions

According to the data generated by the Minitab software, the parameter with the greatest impact in nanoparticle size was the amount of co-polymer (PLGA) used. Increasing the concentration of prednisolone (API) had the most significant effect in increasing entrapment efficiency within the nanoparticle formulation. Also, the homogenization time had minimal effect on the entrapment efficiency. The optimized prednisolone nanoparticle obtained displayed a particle size of 294.5 ± 1.8 nm with surface charge of 5.6 ± 1.8 nm. The NP-loaded contact lenses were then compared to unloaded NP contact lenses. Unloaded contact lenses had a slightly better hydration of 2% difference when compared to nanoparticle-loaded contact lenses. All contact lenses showed good surface wettability. Increase in NP concentration gradually decreased the transmission by 8% when compared to unloaded contact lenses. Drug release studies over 24 h showed that nanoparticle-incorporated contact lenses displayed a slow and prolonged drug release.

## Figures and Tables

**Table 1:** Minitab batch composition. The four independent variables studied are; poly dl-lactic-*co*-glycolic acid (PLGA), polyvinyl alcohol (PVA), amount of prednisolone (API) concentrations and homogenization time (*t*); a partial factorial design with one center point was selected.

StdOrder	RunOrder	CenterPt	Blocks	PLGA % *w*/*v*	PVA % *w*/*v*	API mg/mL	*t* (min)	Size Avg. (*n* = 3)	Zeta Avg. (*n* = 3)
1	1	1	1	0.1	4.0	0.5	10	751.2 ± 126.5	3.1 ± 0.1
2	2	1	1	0.4	4.0	0.5	10	710.6 ± 174.4	2.9 ± 0.4
9	3	1	1	0.1	4.0	0.5	20	678.2 ± 57.2	2.6 ± 0.8
8	4	1	1	0.4	7.5	2.0	10	556.7 ± 29.0	3.0 ± 0.3
3	5	1	1	0.1	7.5	0.5	10	768.3 ± 112.9	2.0 ± 0.5
11	6	1	1	0.1	7.5	0.5	20	671.9 ± 43.3	3.7 ± 0.2
10	7	1	1	0.4	4.0	0.5	20	668.4 ± 75.7	4.1 ± 1.3
12	8	1	1	0.4	7.5	0.5	20	555.9 ± 25.7	0.8 ± 0.2
7	9	1	1	0.1	7.5	2.0	10	743.7 ± 124.5	3.0 ± 0.3
6	10	1	1	0.4	4.0	2.0	10	708.9 ± 104.0	0.6 ± 0.6
16	11	1	1	0.4	7.5	2.0	20	538.8 ± 45.8	1.0 ± 0.2
15	12	1	1	0.1	7.5	2.0	20	818.3 ± 124.7	1.9 ± 0.1
14	13	1	1	0.4	4.0	2.0	20	653.5 ± 39.0	0.7 ± 0.1
4	14	1	1	0.4	7.5	0.5	10	734.3 ± 78.1	2.1 ± 0.3
5	15	1	1	0.1	4.0	2.0	10	640.2 ± 46.6	0.2 ± 0.3
13	16	1	1	0.1	4.0	2.0	20	692.1 ± 40.6	2.5 ± 0.3
